# Potential New Non-Invasive Therapy Using Artificial Oxygen Carriers for Pre-Eclampsia

**DOI:** 10.3390/jfb8030032

**Published:** 2017-07-30

**Authors:** Hidenobu Ohta, Maiko Kaga, Heng Li, Hiromi Sakai, Kunihiro Okamura, Nobuo Yaegashi

**Affiliations:** 1 Department of Psychophysiology, National Institute of Mental Health, National Center of Neurology and Psychiatry, Kodaira, Tokyo 187-8553, Japan; 2 Department of Psychiatry, Asai Hospital, Togane, Chiba 283-0042, Japan; 3 Department of Pediatrics, National Hospital Organization Sendai Medical Center, Miyagino-Ku, Sendai, Miyagi 983-0045, Japan; maikokaga@med.tohoku.ac.jp; 4 Department of Mental Retardation and Birth Defect Research, National Institute of Neuroscience, National Center of Neurology and Psychiatry, Kodaira, Tokyo 187-8553, Japan; yinzhidr@ncnp.go.jp; 5 Department of Chemistry, Faculty of Medicine, School of Medicine, Nara Medical University, Kashihara, Nara 634-8521, Japan; hirosakai@naramed-u.ac.jp; 6 Tohoku Kosai Hospital, Aoba-ku, Sendai, Miyagi 980-0803, Japan; okamura@tohokukosai.com; 7 Department of Obstetrics and Gynecology, Tohoku University Hospital, Aoba-ku, Sendai, Miyagi 980-8574, Japan; yaegashi@med.tohoku.ac.jp

**Keywords:** pre-eclampsia, hemoglobin vesicle, hypoxic condition, placenta, fetus, brain damage

## Abstract

The molecular mechanisms of pre-eclampsia are being increasingly clarified in animals and humans. With the uncovering of these mechanisms, preventive therapy strategies using chronic infusion of adrenomedullin, vascular endothelial growth factor-121 (VEGF-121), losartan, and sildenafil have been proposed to block narrow spiral artery formation in the placenta by suppressing related possible factors for pre-eclampsia. However, although such preventive treatments have been partly successful, they have failed in ameliorating fetal growth restriction and carry the risk of possible side-effects of drugs on pregnant mothers. In this study, we attempted to develop a new symptomatic treatment for pre-eclampsia by directly rescuing placental ischemia with artificial oxygen carriers (hemoglobin vesicles: HbV) since previous data indicate that placental ischemia/hypoxia may alone be sufficient to lead to pre-eclampsia through up-regulation of sFlt-1, one of the main candidate molecules for the cause of pre-eclampsia. Using a rat model, the present study demonstrated that a simple treatment using hemoglobin vesicles for placental ischemia rescues placental and fetal hypoxia, leading to appropriate fetal growth. The present study is the first to demonstrate hemoglobin vesicles successfully decreasing maternal plasma levels of sFlt-1 and ameliorating fetal growth restriction in the pre-eclampsia rat model (*p* < 0.05, one-way ANOVA). In future, chronic infusion of hemoglobin vesicles could be a potential effective and noninvasive therapy for delaying or even alleviating the need for Caesarean sections in pre-eclampsia.

## 1. Introduction

Pre-eclampsia influences approximately 5% of all pregnant women and remains a major cause of maternal and fetal morbidity and mortality [1,2,3]. The hypertension related to pre-eclampsia develops during pregnancy and remits after delivery, suggesting that the placenta is the most likely origin of this disease. The pathophysiology is based on insufficient trophoblast invasion, resulting in incomplete narrow placental spiral artery remodeling. Placental insufficiency, which limits the maternal-fetal exchange of gas and nutrients, leads to fetal intrauterine growth restriction [4,5,6].

The molecular mechanism of pre-eclampsia is being increasingly clarified, and a hypothesis of pre-eclampsia causing endothelial dysfunction of maternal blood vessels has also been proposed: excess placental secretion of sFlt-1 (soluble fms-like tyrosine kinase, also known as soluble vascular endothelial growth factor (VEGF) receptor 1) and sEng (soluble Endoglin) (two endogenous circulating anti-angiogenic proteins) inhibit VEGF and transform growth factor (TGF)-β1 signaling, respectively, in the vasculature, resulting in endothelial cell dysfunction, involving decreased prostacyclin, nitric oxide (NO) production and release of procoagulant proteins. The release of sFlt1 and sEng and other inflammatory mediators seems to be induced by maternal factors such as angiotensin II type I receptor activating autoantibodies (AT1-AA), immunologic factors, and oxidative stress [7]. These molecular mechanisms are considered to induce insufficient trophoblast invasion into the decidua portion of the placenta, resulting in incomplete, narrow placental spiral artery remodeling, leading to hypoxic conditions in the placenta. The exact mechanisms responsible for the pathogenesis of pre-eclampsia, however, still remain unclear and further research on its pathogenic mechanisms and clinical treatments is expected.

Instead of trying to develop preventive treatments similar to those in previous studies [7,8,9,10], which focus on blocking narrow spiral artery formation by suppressing all related possible factors for pre-eclampsia, we decided to take a different strategy by developing a new symptomatic therapy using an artificial oxygen carrier (hemoglobin vesicles: HbV). In this therapy, we directly rescue placental and fetal hypoxia with artificial oxygen carriers, which pass through the narrow spiral arteries already developed in pre-eclampsia and convey oxygen to the hypoxic placental tissues. This new therapy is also expected to suppress sFlt-1 secretion, which has been hypothesized to be induced by hypoxic conditions in the placental tissue and further develops pre-eclampsia [5].

Possible applications of artificial oxygen carriers for treatment of hypoxia-induced pathology such as brain ischemia have been recently suggested by animal models. One advantage of hemoglobin vesicles over usual red blood cells is that they can provide an effective supply of oxygen even to the pathogenic narrow capillary vessels because of the carriers’ nano-scale size and their absence of blood-type antigens [11,12]. The same strategy can be also applied to the placental spiral arteries in pre-eclampsia, in which artificial oxygen carriers treat fetal hypoxia by effectively supplying oxygen, passing through pathogenic narrow spiral arteries in the placenta induced or already established by pre-eclampsia.

To test this hypothesis, we used a rat pre-eclampsia model in which continuous administration of the NO synthetase inhibitor, NG-nitro-l-arginine methyl ester (l-NAME), has been confirmed to induce narrow spiral artery formation, leading to maternal hypertension, placental apoptosis, increased serum tumor necrosis factor (TNF)-α, and fetal hypoxia - all of which have been implicated as being pathophysiological features of pre-eclampsia [13,14]. In this model, we hypothesize that continuous intravenous administration of hemoglobin vesicle (HbV), when added to an l-NAME-induced state of chronic NO inhibition during pregnancy, will treat placental and fetal hypoxia and improve fetal development. In this study, we applied to the pre-eclampsia model rat a daily HbV dose of 200 mg/kg/day, which has been proven to be the most effective and safe dosage to rescue hypoxic tissues in animal models in previous studies [12,15].

## 2. Materials and Methods

### 2.1. Preparation of Hemoglobin Vesicle (HbV) Suspension

The test fluid, the HbV suspension, was prepared under sterile conditions as reported previously [16,17]. Human Hb was purified from outdated donated blood provided by the Japanese Red Cross Society (Tokyo, Japan) through pasteurization and nanofiltration. The encapsulated Hb contained pyridoxal 5-phosphate (Aldrich Chemical Co., Milwaukee, WI, USA) as an allosteric effector. The lipid bilayer was composed of a mixture of 1,2-dipalmitoyl-sn-glycero-3-phosphatidylcholine, cholesterol, 1,5-O-dihexadecyl-N-succynyl-l-glutamate (Nippon Fine Chemicals Co., Osaka, Japan), and 1,2-distearoyl-sn-glycero-3-phosphatidylethanolamine-N-PEG5000 (NOF Co., Tokyo, Japan) at a molar ratio of 5:4:1:0.03. HbVs were suspended in a physiological salt solution and deoxygenated with bubbling N_2_ for storage [18]. The physicochemical parameters of the HbV are as follows: particle diameter, 252 ± 53 nm; [Hb], 10 g/dL; [lipids], 6–7 g/dL; and oxygen affinity, P_50_, 25–28 Torr.

### 2.2. Animals and Experimental Procedures

Eight-week-old female Wistar rats were housed with food and water ad libitum in a temperature controlled room (23 °C) on a 12:12-h light/dark cycle. After one week of habituation, the 9-week-old female rats were mated overnight during the pro-estrus period and timed pregnant rats (vaginal smear positive, gestational day 0 (G0); term, G22) were used for the following experiments. At G10, rats were anesthetized by isoflurane inhalation and then pentobarbital sodium i.p. and a catheter for drug infusion was surgically secured in the internal jugular vein in all the rats. Drug infusions were performed between 13:00 and 14:00 h (local time), that is, between ZT5 and ZT6, where ZT is Zeitgeber time (light on at ZT0 and off at ZT12). Animal care and use were reviewed and approved by the Committee for Tohoku University (approval#2010-73) and the National Center of Neurology and Psychiatry (approval#2011023), and all procedures were performed in accordance with the approved guidelines.

## 3. The Safety and Placental Transfer of Hemoglobin Vesicles (HbV) to Rat Fetus in Pregnancy [15]

**Figure 1:**
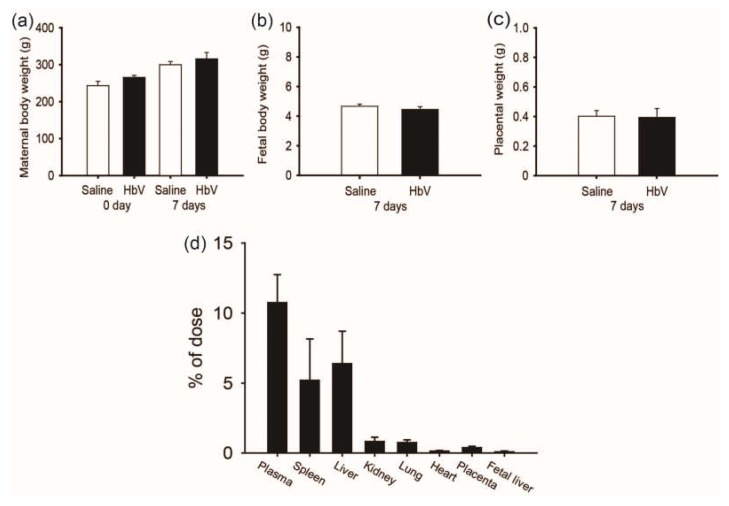
The safety and placental transfer of hemoglobin vesicles (HbV) to fetus in pregnancy. (**a**) Time course of the gains in body weights of pregnant mother rats before and after daily repeated infusions (DRI) of HbV or saline for 7 days at a dose rate of 2 mL/kg/day (*n* = 5 for each group; value: average ± s.d.). The weights of fetuses (**b**) and the placentas (**c**) after 7 days’ DRI of HbV or saline (*n* = 5 for each group; value: average ± s.d.). (**d**) Tissue distributions of 125I-HbV at 12 h after administration to pregnant rats. Rats received a single injection of 125I-HbV from the tail vein at a dose of 1400 mg/kg. Twelve hours after injection, each organ was collected. (*n* = 5 for each group; value: average ± s.d.).

A rat placental model was chosen due to its broad application in the study of placental development and reproduction. As the period for HbV application in pregnant rats, we selected the middle-late stage of pregnancy, in which the rat chrioallantoic placenta starts differentiating into two distinct zones: the maternal-facing junctional zone for the endocrine/invasive functions and the adjacent fetal-facing labyrinthine zone responsible for maternal-fetal exchange [19]. Since the rat placental barrier is not fully differentiated until the latter period of pregnancy, the last trimester was chosen for this study of HbV infusion. From gestational day 15 (G15) to G21, HbV was administered via the catheter in the internal jugular vein at a daily dose of 2 mL/kg, which had been previously proposed as a possible dosage for treatment of acute brain ischemia in adult rat models [12,13,14,15,16,17,18,19,20,21]. Our safety study of HbV demonstrated that daily repeated infusions (DRI) of HbV had no obvious side effects on the pregnant mother or on fetal development (Figure 1a–c). Vital signs, plasma clinical chemistry, and blood gas parameters of mother rats were overall normal after DRI of HbV. In addition, maternal/fetal transfer of HbV was limited to the placenta and HbV was not detected in the fetuses. Histopathological examination with human hemoglobin antibody indicated HbV accumulation in the maternal spleen, liver, kidney, and placenta, but not in the fetuses. These results were reconfirmed by a pharmacokinetic study using ^125^I-labeled HbV (Figure 1d).

## 4. The Rat Pre-Eclampsia Model and Hemoglobin Vesicles (HbV)

### 4.1. Preparation of the Rat Pre-Eclampsia Model by l-NAME Infusion into Mother Rats

During 7 consecutive days between gestational day 14 (G14) and G20, the rats received either saline (*n* = 5), 50 mg/day of l-NAME (Sigma, St. Louis, MO, USA) (*n* = 5), or 50 mg/day of l-NAME + 200 mg/kg/day of hemoglobin vesicle (HbV) (*n* = 5) via the catheter. For the l-NAME + HbV group, HbV was infused at a dose rate of 2 mL/kg body weight with an injection rate of 1 mL/min. The total volume of HbV infused into each rat over 7 days reached 14 mL/kg, which was equal to 25% of the actual blood volume of the rat (56 mL/kg). The total infused HbV was calculated to be 1400 mg Hb/kg. The maternal blood pressure was also measured from G14 to G21 between ZT5 and ZT6 once a day. At G21, one day before term, the pregnant rats were sacrificed, and the fetuses, placenta, and maternal blood were sampled for assessment of placental and fetal hypoxia, placental and fetal weights, and plasma sFlt-1 and sEng, markers of pre-eclampsia between ZT5 and ZT6.

### 4.2. Change in Maternal Blood Pressure during Gestational Period

**Figure 2:**
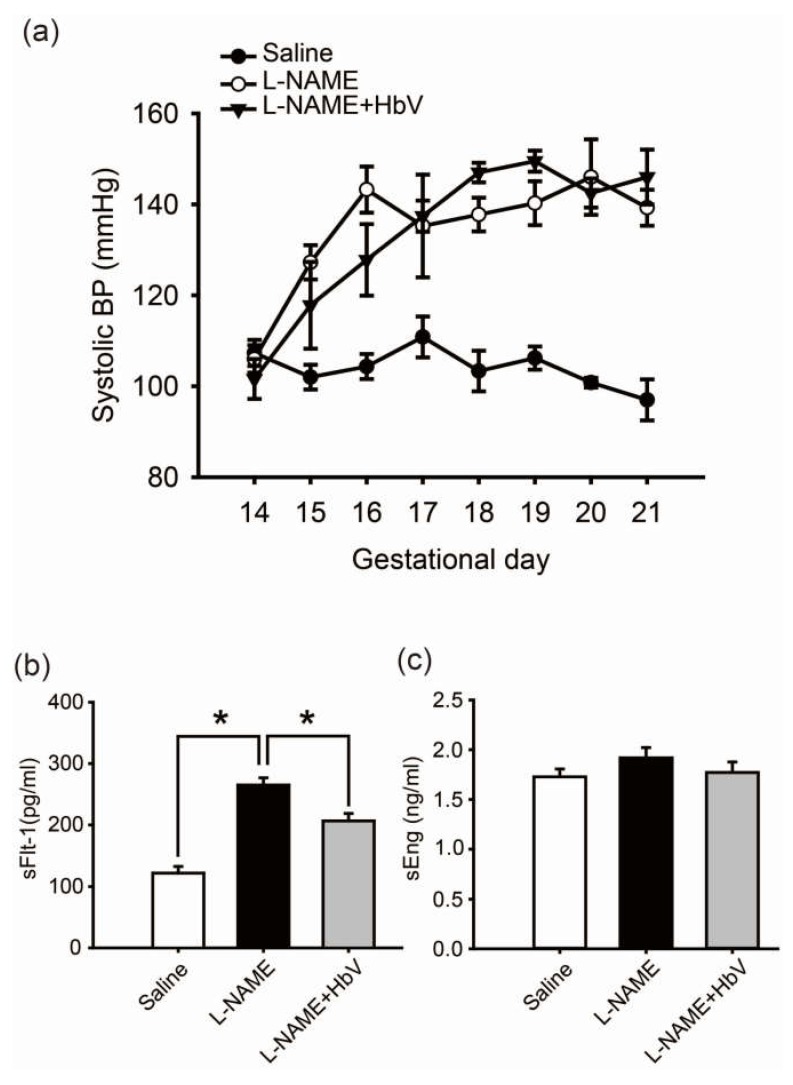
Effects of hemoglobin vesicle (HbV) infusion on maternal blood pressure and sFlt-1/sEng production in pregnant rats. (**a**) Chronological changes in systolic blood pressure during pregnancy in saline (control), NG-nitro-l-arginine methyl ester (l-NAME)-only treated and l-NAME + HbV treated pregnant rats. Closed circles, saline control pregnant rats; open circles, l-NAME-only treated pregnant rats; closed triangles, l-NAME + HbV treated pregnant rats. The data for each group are expressed as mean values ± s.e. (*n* = 5). Significant difference between control and l-NAME-only treated rats or l-NAME + HbV treated rats was observed (* *p* < 0.05, two-way ANOVA, Dunette); (**b**)The plasma sFlt-1 levels in l-NAME-only treated rats were significantly higher compared with those in saline control pregnant rats or l-NAME + HbV treated rats; (**c**)There was no statistical significance in the plasma sEng among the three groups. Data are expressed as mean ± s.e. (* *p* < 0.05, one-way ANOVA, Dunette).

In the rat pre-eclampsia model, chronological changes in systolic blood pressure (SBP) levels were measured by a tail-cuff method using a Model MK-2000ST recorder (Muromachi Kikai Co., Ltd., Tokyo, Japan) once every day (Figure 2a) [22]. Fifty mg/day of l-NAME was intravenously infused through a catheter in the jugular vein for 7 consecutive days between gestational day 14 (G14) and G21. The mean SBP at G14 (prior to the start of this experiment) was 107.3 ± 2.9 mmHg (mean ± s.e.) in the control pregnant rats and 106 ± 6.8 mmHg in the group of pregnant rats awaiting l-NAME treatment. Once l-NAME was administered, the mean SBP of the pregnant rats in this treated group gradually increased by day 16 of pregnancy. This elevation in SBP was maintained until day 21 of pregnancy. A similar increase in mean SBP level was also observed in the group of pregnant rats which received both 50 mg/day of l-NAME and 200 mg/kg/day of hemoglobin vesicles (the l-NAME + HbV treated group). No statistical difference in SBP between the l-NAME-only treated and l-NAME + HbV treated groups was observed between G18 and G21.

### 4.3. Hemoglobin Vesicles (HbV) Decreased sFlt-1 Plasma Levels in Maternal Blood

We also investigated the effect of HbV on production of sFlt-1 and sEng, candidate molecules for the cause of pre-eclampsia, in the placenta by comparing by ELISA the plasma levels of sFlt-1 and sEng among the three groups: saline (control), l-NAME, and l-NAME + HbV treated groups. Pregnant control animals had a significantly lower level of sFlt-1 (122 pg/mL ± 11) than l-NAME-only (265 pg/mL ± 12) and l-NAME + HbV (207 pg/mL ± 12) treated animals during pregnancy (Figure 2b, *p* < 0.05, one-way ANOVA, Dunette). In addition, l-NAME-only treated animals had a higher level of sFlt-1 than l-NAME + HbV treated animals. However, there was no statistically significant difference in the plasma sEng between the three groups (Figure 2c, one-way ANOVA, Dunette). This data suggest that HbV contributed to a decrease in sFlt-1 production by reducing hypoxic conditions in the placenta. Maternal blood pressure (BP), however, continued to remain high as l-NAME, a nitric oxide synthase (NOS) inhibitor which directly regulates nitric-oxide (NO) mediated vasoconstriction, is downstream of sFlt-1 and sEng.

### 4.4. Hemoglobin Vesicles (HbV) Improved Placental Hypoxic Conditions

**Figure 3:**
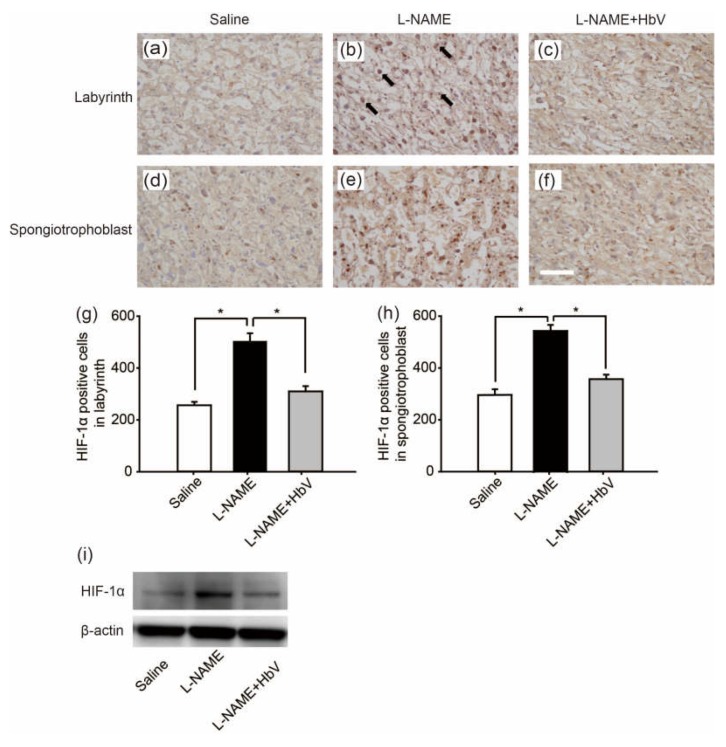
Effects of hemoglobin vesicle (HbV) infusion on placental hypoxia in pregnant rats. Hypoxic-inducible factor 1α (HIF-1α) (brown signal) shows stronger signal activity in the labyrinth and spongiotrophoblast of the l-NAME-only treated group (**b,e**) compared with that of the saline control group (**a**,**d**) or the l-NAME + HbV treated group (**c**,**f**). Arrows in (**b**) indicate representative HIF-1α positive cells (dark brown cells). Scale bar: 300 µm. Quantification of the HIF-1α-positive cells in the labyrinth (**g**) and spongiotrophoblast (**h**). Western blot analysis of HIF-1α in the placental tissues from saline, l-NAME, and l-NAME + HbV treated pregnant rats (**i**).

Next, to confirm the placental hypoxic conditions in the rat pre-eclampsia model, we examined hypoxic-inducible factor 1α (HIF-1α) expression in the placenta. Figure 3a–f shows HIF-1α in the labyrinth (Figure 3a–c) and spongiotrophoblast (Figure 3d–f) in fetuses whose mother rats received continuous infusions of saline (Figure 3a,d), l-NAME (Figure 3b,e), or l-NAME + HbV (Figure 3c,f) from G14 through G21. In the labyrinth, HIF-1α positive cells were increased significantly more in the l-NAME-only treated group (509.8 cells ± 31.1) than in that of the saline (control) (255.8 cells ± 13.4) or l-NAME + HbV treated (309.9 cells ± 20.6) groups (Figure 3g, *p* < 0.05, one-way ANOVA, Dunette). In the spongiotrophoblast, HIF-1α positive cells were increased significantly more in the l-NAME-only treated group (537.3 cells ± 22.8) than in that of the saline (289.3 cells ± 21.4) or l-NAME + HbV treated (349.2 cells ± 16.8) groups (Figure 3h, *p* < 0.05, one-way ANOVA, Dunette). Western blot also showed that the HIF-1α expression in the whole placenta was significantly higher in the l-NAME-only treated group than that of the saline or l-NAME + HbV treated groups (Figure 3i). The data indicate that HbV treatment on pregnant mother rats rescued hypoxic conditions in the placenta.

### 4.5. Effects of Hemoglobin Vesicle (HbV) Infusion on Fetal Brain and Body Growth under Hypoxic Conditions

#### 4.5.1. Evaluation of Fetal Hypoxic Conditions by Bioluminescence

**Figure 4:**
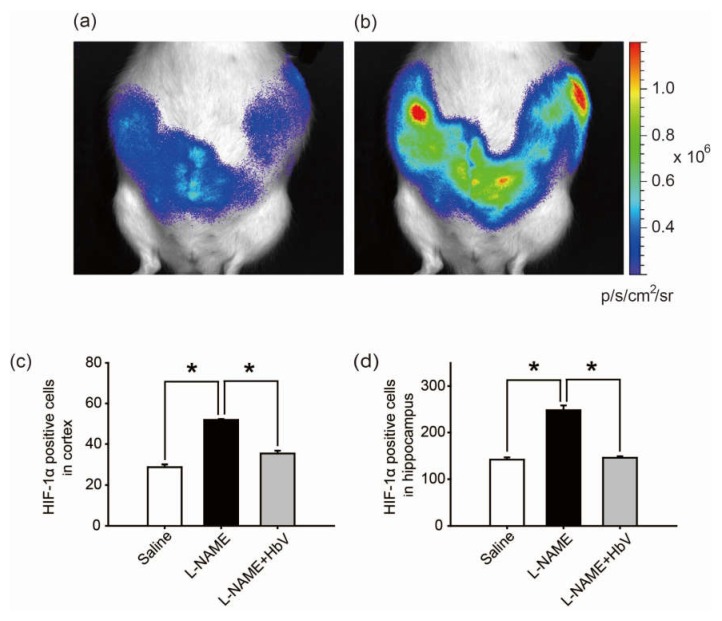
Effects of hemoglobin vesicle (HbV) infusion on fetal hypoxia. Photon flux (p/s/cm^2^/sr) from the heterozygous Rosa 26::luc fetuses is displayed according to the scale bar at the right side. Compared to the basal state of saline injection (**a**), 60 s exposures show that light emission increased after an acute HbV injection (**b**). Quantification of the HIF-1α-positive cells in the cortex (**c**) and hippocampus (**d**). Data are expressed as mean ± s.e. (* *p* < 0.05, one-way ANOVA, Dunette). Maternal l-NAME injection induced fetal brain hypoxia, as indicated by more extensive HIF-1α positive staining at the cortex and hippocampus in fetal brain compared to the same areas of fetal brains from saline or l-NAME + HbV treated mothers. Note that HbV attenuated l-NAME-induced fetal brain hypoxia.

Finally, we investigated whether HbV can rescue fetal hypoxia and improve fetal development. To visually evaluate fetal hypoxia, the hypoxic levels of Rosa 26::Luc rat fetuses in the pregnant uterus were examined by bioluminescence in vivo (Figure 4a,b) [23,24]. Rosa 26 gene is known to express itself ubiquitously in all tissues. Thus, the luciferase gene, which is fused to the endogenous Rosa 26 gene promoter, can be monitored throughout the whole body of Rosa 26::Luc rats by in vivo imaging. Rosa 26::Luc rat fetuses were produced by mating a wild-type female Wistar rat with a male homozygous Rosa 26::Luc rat, resulting in bioluminescence being detectable only from heterozygous Rosa 26::Luc rat fetuses and not from the mother rat. Since firefly luciferase requires oxygen, adenosine triphosphate (ATP), and luciferin to emit light, the bioluminescence of Rosa 26::Luc rat fetuses was expected to be elevated by increased oxygen supply to the placentas by HbV. The bioluminescence of the fetuses was assessed after continuous syringe pump infusion of luciferin (0.01 M luciferin, 0.5 mL/h) into the pregnant rats through the catheter in the jugular vein. The bioluminescence levels of the fetuses of l-NAME-only treated mother rats became 1.71 times brighter after acute HbV injections of 0.6 mL, which is equivalent to the volume of a daily HbV injection, than bioluminescence levels of the fetuses after acute saline injections of 0.6 mL (*n* = 5, *p* < 0.05, one-way ANOVA). Figure 4b demonstrates representative data of the increased bioluminescence levels of the fetuses after an HbV injection, indicating that l-NAME-induced fetal hypoxia was rescued by an acute HbV injection.

#### 4.5.2. Evaluation of Hypoxic Conditions of the Fetal Brain

The hypoxic conditions in the fetus were evaluated by HIF-1α expression in the fetal brain. In the cortex, HIF-1α positive cells were significantly increased in the l-NAME-only treated group (51.9 cells ± 0.4) compared to that in the saline group (28.7 cells ± 1.3) or the l-NAME + HbV treated group (35.4 cells ± 1.3) (Figure 4c, *p* < 0.05, one-way ANOVA, Dunette). In the hippocampus, HIF-1α positive cells were significantly increased in the l-NAME-only treated group (248.0 cells ± 10.3) in comparison to that in the saline group (141.1 cells ± 5.0) or the l-NAME + HbV treated group (145.5 cells ± 3.3) (Figure 4d, *p* < 0.05, one-way ANOVA, Dunette). The data indicate that HbV treatment to pregnant mother rats rescued hypoxic conditions in the fetal brain.

#### 4.5.3. Hemoglobin Vesicles (HbV) Protected Fetal Brain Apoptotic Damage from Hypoxia

**Figure 5:**
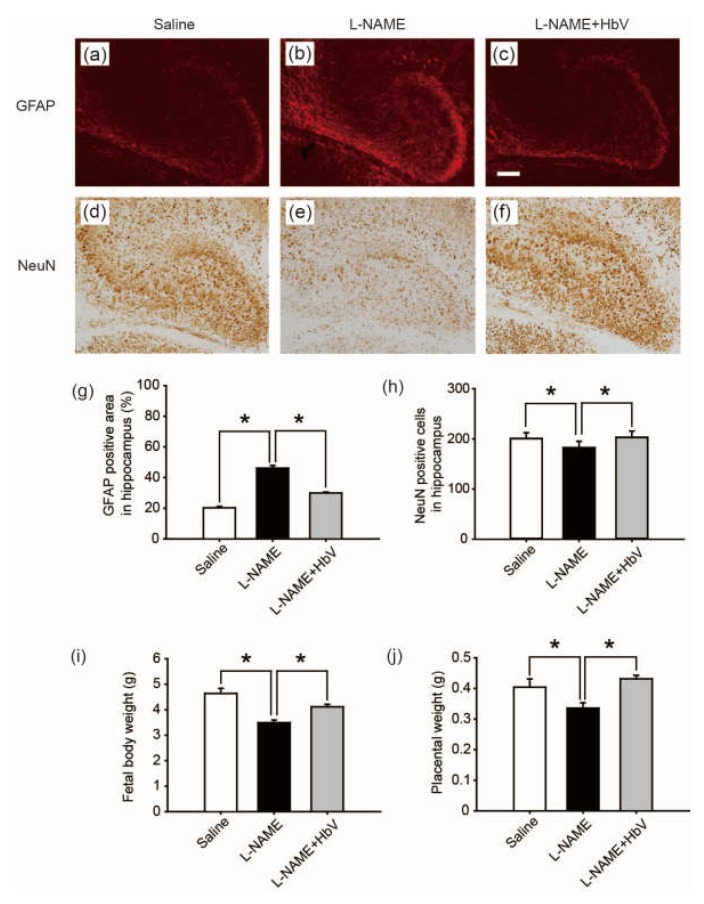
Effects of hemoglobin vesicle (HbV) infusion on fetal brain and body growth under hypoxic conditions. The hippocampus of G21 fetuses from the pregnant rats treated with saline (**a**, *n* = 5), l-NAME (**b**, *n* = 5), or l-NAME + HbV (**c**, *n* = 5) are shown. The glial fibrillary acidic protein (GFAP) positive staining in the hippocampus of fetuses (**b**) from l-NAME treated mothers was stronger compared with those from the saline treated mothers (**a**) or l-NAME + HbV treated mothers (**c**). This indicated that maternal l-NAME injection induced fetal brain astrogliosis and HbV reduced the l-NAME-induced fetal brain damage. Quantification of the GFAP-positive area in the hippocampus (**g**). Data are expressed as mean ± s.e. (* *p* < 0.05, one-way ANOVA, Dunette). The number of NeuN-positive cells in the hippocampus of fetuses (**e**) from l-NAME treated mothers was smaller compared with that in the saline treated mothers (**d**) or l-NAME + HbV treated mothers (**f**). This indicated that maternal l-NAME injection induced neural damage in the fetal brain while HbV reduced the l-NAME-induced fetal brain damage. Quantification of the NeuN-positive cells in the hippocampus (**h**). Data are expressed as mean ± s.e. (* *p* < 0.05, one-way ANOVA, Dunette). The weight gains of rat fetuses (**i**) and the placentas (**j**) after chronic HbV or saline infusion at a dose rate of 2 mL/kg/day (*n* = 5 for each group; value: average ± s.e.) for 7 days. Note that HbV rescued the fetal and placental weights in l-NAME + HbV treated groups compared those in l-NAME-only treated groups. Scale bar: 100 µm.

To evaluate fetal brain apoptotic damage from hypoxia, we examined reactive astrogliosis using glial fibrillary acidic protein (GFAP) and NeuN immunostaining [25,26,27]. GFAP-positive areas in the cortex showed no statistical difference among the three groups: saline, l-NAME-only, and l-NAME + HbV treated groups (one-way ANOVA, Dunette). In contrast, GFAP-positive areas in the dentate regions of the hippocampus showed that the l-NAME-only treated group (46.1 ± 1.7%, Figure 5b) induced a significant increase in GFAP expression compared to that of the saline group (20.3 ± 0.9%, Figure 5a) or the l-NAME + HbV (30.0 ± 0.6%, Figure 5c) treated group (one-way ANOVA, Dunette, *p* < 0.05, Figure 5g), indicating that HbV treatment rescued fetal hippocampus apoptotic damage from hypoxia. Likewise, NeuN-positive cells in the cortex showed no statistical difference among the three groups (one-way ANOVA, Dunette). In contrast, NeuN-positive cells in the dentate regions of the hippocampus showed that the l-NAME-only treated group (180.3 cells ± 12.7, Figure 5e) induced a significant decrease in NeuN-positive cells compared to that of the saline group (198.2 cells ± 11.7, Figure 5d) and the l-NAME + HbV (200.6 cells ± 12.3, Figure 5f) treated group (one-way ANOVA, Dunette, *p* < 0.05, Figure 5h), also indicating that HbV treatment rescued fetal hippocampus apoptotic damage from hypoxia.

#### 4.5.4. Hemoglobin Vesicles (HbV) Improved Fetal Body Growth under Hypoxic Conditions

We also examined the effects of HbV on fetal body growth by assessing weight gain. Body weights of fetuses in the l-NAME-only treated group (3.5 g ± 0.1) were significantly lower than those of the fetuses in the saline treated group (4.6 g ± 0.2). HbV infusion reversed l-NAME-induced reduction of average fetal body weight (4.1 g ± 0.1) in the l-NAME + HbV treated group (Figure 5i). The placental weights (0.34 g ± 0.02) of l-NAME-only treated mother rats were significantly lower than those of saline (0.40 g ± 0.03) or l-NAME + HbV treated mother rats (0.43 g ± 0.01) (Figure 5j). These data indicate that HbV treatment on l-NAME treated pregnant mother rats ameliorated fetal growth restriction.

## 5. Discussion

The present study reports two novel findings regarding the effects of artificial oxygen carriers (hemoglobin vesicles: HbV) in the rat model of pre-eclampsia. Firstly, we are the first to report that HbV ameliorates the fetal growth restriction and brain apoptotic damage in the pre-eclampsia rat model. This is in contrast to previous therapy strategies using chronic infusion of adrenomedullin, VEGF-121, and sildenafil, which failed to rescue the fetal body weight gain [8,9,10]. The different results between the present and previous studies may relate to the extent that each therapy rescues hypoxic conditions in the placenta, which also leads to reduction of fetal hypoxia. As shown in Figure 3 and Figure 4, HIF-1α expressions in both the placenta and fetal brain tissues were decreased by HbV infusion, indicating that HbV rescued fetal hypoxia by increasing oxygen supply through the placenta. This is also supported by in vivo imaging of Rosa 26::luc fetuses, in which the bioluminescence of fetuses is increased by acute HbV injections, indicating that fetal hypoxic conditions are rescued by oxygen supply provided by HbV (Figure 4a,b). In addition, our data indicate that brain injury, reactive astrogliosis, in fetal brain tissue due to hypoxia was prevented by chronic HbV infusion (Figure 5a–h).

Second, we also found that the serum level of sFlt-1 was decreased in the l-NAME + HbV treated group compared to that of the l-NAME-only treated group. This is consistent with previous reports stating that placental ischemia/hypoxia results in elevated circulating levels of sFlt-1 [4] since chronic HbV infusion seems to have directly rescued placental hypoxia in the present study. Our data also matched the recent data in the same rat pre-eclampsia model, in which chronic l-NAME infusions increased sFlt-1 but induced no change in sEng in pregnant rats [28,29]. The data indicates a possible new treatment using HbV for pre-eclampsia, in which excess placental secretion of sFlt-1 and sEng inhibits VEGF and TGF-β1 signaling, respectively, in the vasculature, resulting in decreased endothelial NO production. Future studies, such as applying HbV to another pre-eclampsia animal model induced by sFlt-1 and sEng agonists, or systemic measurement of all pre-eclampsia-related substances after HbV treatment, would be required to reconfirm the effects of HbV on the pre-eclampsia mechanism.

Interestingly, in humans, minimum capillary diameters have been reported to be around 8 µm, approximately equal in size to human red blood cells [30]. Previous studies have indicated that, even among different species, all mammals have similar capillary diameters since their peripheral tissues have the same physiological properties necessary for exchanging gas, nutrients, and metabolic wastes between capillary vessels [31]. These data support the possibility that application of 250 nm-diameter HbV will effectively treat pre-eclampsia in humans by supplying oxygen through narrow placental vessels induced by pre-eclampsia. Although further investigations are required to elucidate whether the same effects would occur in humans, our results are the first to describe the treatment effect of HbV on fetuses and placentas in a pre-eclampsia rat model and have also provided important information for possible HbV clinical applications for addressing fetal hypoxic conditions induced by a pathogenic placenta during pregnancy. In the future, chronic infusion of HbV could be a potentially effective noninvasive therapy for delaying or even alleviating the need for Caesarean sections, the last-resort therapy for pre-eclampsia [32].

One concern in this study warrants consideration: that is the difficulty of developing a treatment for maternal hypertension from pre-eclampisa in the present experimental design. Since l-NAME is expected to control maternal vasoconstriction at the last stage of the cascade of the vasoconstriction mechanism [33,34,35], no other drug, including hemoglobin vesicles (HbV), is likely to release vasoconstriction and decrease maternal hypertension. To investigate whether HbV actually can also decrease maternal hypertension from pre-eclampsia, it is necessary to apply HbV to another pre-eclampisa animal model without using l-NAME.

## 6. Conclusions

The present study demonstrated that a simple treatment using hemoglobin vesicles (HbV) for placental ischemia rescues placental and fetal hypoxia in a pre-eclampsia rat model, leading to appropriate fetal growth. The study also demonstrated HbV successfully decreasing maternal plasma levels of sFlt-1 and ameliorating fetal growth restriction. Human clinical studies will be required to further evaluate the chronic infusion of HbV as a potential effective, noninvasive, and safe therapy for pre-eclampsia.

## References

[1] Sibai B., Dekker G., Kupferminc M. (2005). Pre-eclampsia. Lancet.

[2] Bibbins-Domingo K., Grossman D.C., Curry S.J., Barry M.J., Davidson K.W., Doubeni C.A., Epling J.W., Kemper A.R., Krist A.H., Kurth A.E. (2017). Screening for preeclampsia: US Preventive Services Task Force recommendation statement. JAMA..

[3] Henderson J.T., Thompson J.H., Burda B.U., Cantor A. (2017). Preeclampsia screening: Evidence report and systematic review for the US Preventive Services Task Force. JAMA..

[4] Myatt L., Webster R.P. (2009). Vascular biology of preeclampsia. J. Thromb. Haemost..

[5] Powe C.E., Levine R.J., Karumanchi S.A. (2011). Preeclampsia, a disease of the maternal endothelium: The role of antiangiogenic factors and implications for later cardiovascular disease. Circulation.

[6] Cotechini T., Komisarenko M., Sperou A., Macdonald-Goodfellow S., Adams M.A., Graham C.H. (2014). Inflammation in rat pregnancy inhibits spiral artery remodeling leading to fetal growth restriction and features of preeclampsia. J. Exp. Med..

[7] Zhou C.C., Zhang Y., Irani R.A., Zhang H., Mi T., Popek E.J., Hicks M.J., Ramin S.M., Kellems R.E., Xia Y. (2008). Angiotensin receptor agonistic autoantibodies induce pre-eclampsia in pregnant mice. Nat. Med..

[8] Witlin A.G., Gangula P.R., Wimalawansa S.J., Grafe M., Grady J.J., Yallampalli C. (2003). Adrenomedullin requires an intact nitric oxide system to function as an endogenous vasodilator in rat gestation. Hypertens Pregnancy.

[9] Gilbert J.S., Verzwyvelt J., Colson D., Arany M., Karumanchi S.A., Granger J.P. (2010). Recombinant vascular endothelial growth factor 121 infusion lowers blood pressure and improves renal function in rats with placental ischemia-induced hypertension. Hypertension.

[10] Herraiz S., Pellicer B., Serra V., Cauli O., Cortijo J., Felipo V., Pellicer A. (2012). Sildenafil citrate improves perinatal outcome in fetuses from pre-eclamptic rats. BJOG.

[11] Chang T.M. (2005). Therapeutic applications of polymeric artificial cells. Nat. Rev. Drug. Discov..

[12] Kawaguchi A.T., Fukumoto D., Haida M., Ogata Y., Yamano M., Tsukada H. (2007). Liposome-encapsulated hemoglobin reduces the size of cerebral infarction in the rat: Evaluation with photochemically induced thrombosis of the middle cerebral artery. Stroke.

[13] Tsukimori K., Komatsu H., Fukushima K., Kaku T., Nakano H., Wake N. (2008). Inhibition of nitric oxide synthetase at mid-gestation in rats is associated with increases in arterial pressure, serum tumor necrosis factor-alpha, and placental apoptosis. Am. J. Hypertens.

[14] Verlohren S., Niehoff M., Hering L., Geusens N., Herse F., Tintu A.N., Plagemann A., LeNoble F., Pijnenborg R., Muller D.N. (2008). Uterine vascular function in a transgenic preeclampsia rat model. Hypertension.

[15] Kaga M., Li H., Ohta H., Taguchi K., Ogaki S., Izumi H., Inagaki M., Tsuchiya S., Okamura K., Otagiri M. (2012). Liposome-encapsulated hemoglobin (hemoglobin-vesicle) is not transferred from mother to fetus at the late stage of pregnancy in the rat model. Life Sci..

[16] Sakai H., Hara H., Yuasa M., Tsai A.G., Takeoka S., Tsuchida E., Intaglietta M. (2000). Molecular dimensions of Hb-based O_2_ carriers determine constriction of resistance arteries and hypertension. Am. J. Physiol. Heart Circ. Physiol..

[17] Sou K., Naito Y., Endo T., Takeoka S., Tsuchida E. (2003). Effective encapsulation of proteins into size-controlled phospholipid vesicles using freeze-thawing and extrusion. Biotechnol. Prog..

[18] Sakai H., Tomiyama K.I., Sou K., Takeoka S., Tsuchida E. (2000). Poly(ethylene glycol)-conjugation and deoxygenation enable long-term preservation of hemoglobin-vesicles as oxygen carriers in a liquid state. Bioconjug. Chem..

[19] Soares M.J. (1997). Molecular mechanisms of placental development. Placental Function and Fetal Nutrition.

[20] Sakai H., Masada Y., Horinouchi H., Yamamoto M., Ikeda E., Takeoka S., Kobayashi K., Tsuchida E. (2004). Hemoglobin-vesicles suspended in recombinant human serum albumin for resuscitation from hemorrhagic shock in anesthetized rats. Crit. Care Med..

[21] Kakehata J., Yamaguchi T., Togashi H., Sakuma I., Otani H., Morimoto Y., Yoshioka M. (2010). Therapeutic potentials of an artificial oxygen-carrier, liposome-encapsulated hemoglobin, for ischemia/reperfusion-induced cerebral dysfunction in rats. J. Pharmacol. Sci..

[22] Kubota Y., Umegaki K., Kagota S., Tanaka N., Nakamura K., Kunitomo M., Shinozuka K. (2006). Evaluation of blood pressure measured by tail-cuff methods (without heating) in spontaneously hypertensive rats. Biol. Pharm. Bull..

[23] Hakamata Y., Murakami T., Kobayashi E. (2006). “Firefly rats” As an organ/cellular source for long-term in vivo bioluminescent imaging. Transplantation.

[24] Tahara Y., Kuroda H., Saito K., Nakajima Y., Kubo Y., Ohnishi N., Seo Y., Otsuka M., Fuse Y., Ohura Y. (2012). In vivo monitoring of peripheral circadian clocks in the mouse. Curr Biol.

[25] Deguchi K., Oguchi K., Takashima S. (1997). Characteristic neuropathology of leukomalacia in extremely low birth weight infants. Pediatr. Neurol..

[26] Back S.A. (2001). Recent advances in human perinatal white matter injury. Prog. Brain. Res..

[27] Cai Z., Pang Y., Lin S., Rhodes P.G. (2003). Differential roles of tumor necrosis factor-alpha and interleukin-1 beta in lipopolysaccharide-induced brain injury in the neonatal rat. Brain. Res..

[28] Ramesar S.V., Mackraj I., Gathiram P., Moodley J. (2011). Sildenafil citrate decreases sFlt-1 and sEng in pregnant l-name treated sprague-dawley rats. Eur. J. Obstet. Gynecol. Reprod. Biol..

[29] Bahtiyar M.O., Buhimschi C., Ravishankar V., Copel J., Norwitz E., Julien S., Guller S., Buhimschi I.A. (2007). Contrasting effects of chronic hypoxia and nitric oxide synthase inhibition on circulating angiogenic factors in a rat model of growth restriction. Am. J. Obstet. Gynecol..

[30] Freitas R.A. (1999). Nanomedicine, Vol. IIA: Biocompatibility.

[31] Harry D., Patton A.F.F., Hille B. (1989). Textbook of Physiology.

[32] Li H., Ohta H., Tahara Y., Nakamura S., Taguchi K., Nakagawa M., Oishi Y., Goto Y., Wada K., Kaga M. (2015). Artificial oxygen carriers rescue placental hypoxia and improve fetal development in the rat pre-eclampsia model. Sci. Rep..

[33] Venkatesha S., Toporsian M., Lam C., Hanai J., Mammoto T., Kim Y.M., Bdolah Y., Lim K.H., Yuan H.T., Libermann T.A. (2006). Soluble endoglin contributes to the pathogenesis of preeclampsia. Nat. Med..

[34] Holwerda K.M., Faas M.M., Van Goor H., Lely A.T. (2013). Gasotransmitters: A solution for the therapeutic dilemma in preeclampsia?. Hypertension.

[35] Jerkic M., Letarte M. (2015). Contribution of oxidative stress to endothelial dysfunction in hereditary hemorrhagic telangiectasia. Front Genet.

